# Ecological Frameworks of Pathogen–Pathogen and Pathogen–Microbiome Interactions Within the Tick Holobiont

**DOI:** 10.3390/pathogens15040440

**Published:** 2026-04-18

**Authors:** Elianne Piloto-Sardiñas, Islay Rodríguez, Huarrisson Azevedo Santos, Patrícia Gonzaga Paulino, Belkis Corona-González, Alejandro Cabezas-Cruz

**Affiliations:** 1 Direction of Animal Health, National Center for Animal and Plant Health, Carretera de Tapaste y Autopista Nacional, Apartado Postal 10, San José de las Lajas 32700, Mayabeque, Cuba; bcorogonz@gmail.com; 2 Tropical Medicine Institute ¨Pedro Kourí¨ (IPK), Autopista Novia del Mediodía, km 6 ½, Marianao 13, La Lisa 11400, La Habana, Cuba; islay@ipk.sld.cu; 3 Department of Epidemiology and Public Health, Federal Rural University of Rio de Janeiro (UFRRJ), BR 465, Km 7, Seropedica, Rio de Janeiro 23890-000, Brazil; huarrisson@yahoo.com.br; 4 Department of Genetics, Universidade Federal Rural do Rio de Janeiro (UFRRJ), Seropedica, Rio de Janeiro 23890-000, Brazil; patgpaulino@gmail.com; 5 ANSES, INRAE, Ecole Nationale Vétérinaire d’Alfort, UMR BIPAR, Laboratoire de Santé Animale, F-94700 Maisons-Alfort, France

**Keywords:** tick holobiont, microbial community assembly, pathogen–pathogen interactions, pathogen–microbiome interactions, vector competence

## Abstract

Ticks harbor complex microbial communities composed of symbionts, commensals, and tick-borne pathogens (TBPs). Together, these microorganisms form the tick holobiont. Within this system, the tick’s physiological architecture structures microbial communities by distributing microorganisms across distinct tissues. This compartmentalization creates spatially distinct ecological niches, which in turn shape how microbial communities assemble and interact. In this review, we integrate ecological theory with current knowledge of tick microbiome research to examine how pathogen–pathogen and pathogen–microbiome interactions emerge within these tissue-structured microbial communities. We first outline how baseline ecological filters, including tick species, developmental stage, tissue identity, vertical transmission, and environmental context, shape the microbiome configuration through community assembly processes. We then examined how TBPs, as high-impact colonizers, can further modify microbial networks by altering host-mediated selective pressures, influencing interaction topology, and reshaping community stability. Based on these observations, we propose a dual selective pressure framework in which (i) baseline ecological structuring processes and (ii) pathogen-associated selective pressures interact to determine the microbial network configuration and functional outcomes within the tick holobiont. These interacting forces may drive shifts in diversity, modularity, keystone taxa emergence, and network resilience, ultimately influencing vector competence. This review frames the microbial communities within the tick holobiont as spatially structured ecological systems shaped by multilevel selective pressures. This conceptual foundation provides a coherent framework for understanding microbial interactions in arthropod vectors and highlights avenues for mechanistic research and microbiome-based strategies to mitigate tick-borne diseases.

## 1. Introduction

Arthropod vectors contribute to ecosystem stability at the macroecological level [1,2]. However, beyond their ecological role in trophic cascades, these organisms also contain a diverse range of microorganisms. The presence and multiplication of pathogenic and non-pathogenic microbes within the vector allows it to be conceptualized as a structured environment that harbors a microbial microecosystem shaped by biotic and abiotic determinants [3].

In any microbial ecosystem, whether soil, water, or a host-associated environment, microorganisms do not exist in isolation, nor are they found as single-species populations [4,5,6]. Therefore, interactions are inherent to the establishment of microbial communities, which can even harbor genotypic and phenotypic variations within the same taxon [7,8,9,10].

Furthermore, established communities constantly face external perturbations such as the arrival of new pathogenic, non-pathogenic, and opportunistic taxa, potentially leading to ecological processes such as competition for space and resources. The resulting balance between cooperative and competitive associations within a microbial community ultimately influences its resilience, productivity, and ability to resist microbial invasion [11,12,13,14]. This ecological framework applies directly to arthropod vectors. The vector can be conceptualized as a holobiont, an integrated ecological unit in which the host tissues and associated microorganisms collectively shape the environment for microbial communities [2,15].

Ticks are obligate hematophagous ectoparasites, and their bodies contain a structured microecosystem: a diverse community of microorganisms, including symbionts, commensals, and pathogens, that coexist in specialized tissues, such as the gut, salivary glands, and ovaries [16,17]. The coevolution between ticks and their associated microorganisms has resulted in intricate networks of interactions that extend beyond simple vector–pathogen relationships to encompass the entire microbial community. Understanding how these interactions shape pathogen colonization, maintenance, and transmission is critical for predicting and managing tick-borne diseases, which pose a growing threat to human and animal health [18,19].

In this review, we applied the fundamental frameworks of microbial interactions, based on evidence from tick microbiome studies, to the specific environment provided by ticks. The first section examines the implications of the tick’s structurally and functionally compartmentalized holobiont architecture for establishing and determining the outcomes of microbial interactions. Subsequently, key aspects of microbial interactions are analyzed within this framework. First, pathogen–pathogen interactions are addressed in the context of establishing co-infections, examining evidence from both simultaneous pathogen detection and assembly patterns within ecological guilds. Second, the general biotic and abiotic factors that shape the tick microbiome are reviewed, establishing an ecological baseline upon which interactions occur. Finally, interactions between pathogens and the non-pathogenic microbial communities are explored. Here, the discussion first distinguishes which of the general factors operate in the absence of selection driven by tick-borne pathogens (TBPs) and then examines how the dual selective pressure imposed by basal factors and replicating pathogens jointly shapes the tick microbiome and their interactions. By integrating these lines of evidence, we seek to understand how the ecological principles that govern microbial communities in macroecosystems operate within the microbial microecosystem contained in the tick, ultimately shaping vector competence and opening new avenues for microbiome-based interventions.

Before proceeding, a terminological clarification is required. Throughout this review, unless otherwise specified, the terms “TBPs” and “pathogens” are used interchangeably to refer to microorganisms that complete their transmission cycle via ticks and cause disease in vertebrate hosts. This distinction becomes particularly relevant in Section 5, where we define the “non-pathogenic microbial community” in relation to TBPs, independent of any pathogenic potential these microorganisms may have outside the vector.

## 2. Implications of the Compartmentalized Tick Holobiont for Microbial Interactions

**Figure 1:**
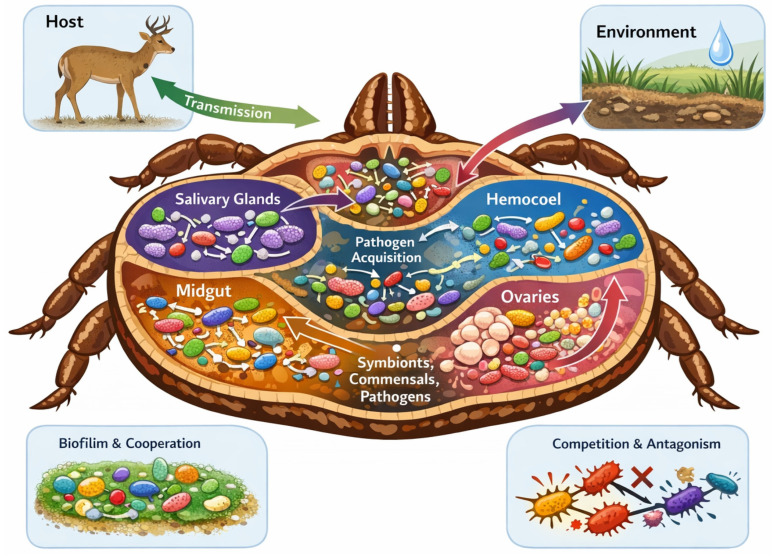
**Spatially structured microbial communities within the tick holobiont.** Schematic representation of tissue-specific microbial communities organized within the physiological architecture of the tick. Distinct tissues (midgut, salivary glands, ovaries, and hemocoel) generate heterogeneous ecological niches characterized by differences in immune activity, nutrient availability, and physicochemical conditions. Each compartment harbors a partially distinct microbial community composed of symbionts, commensals, and TBPs, forming localized interaction networks that may include cooperative processes (e.g., cross-feeding, biofilm formation) and competitive mechanisms (e.g., interference or resource competition). The midgut mediates early microbial interactions during pathogen acquisition from the vertebrate host, whereas salivary glands are central during transmission. Ovarian tissues enable vertical transmission, while environmental and host-derived acquisition contribute to community assembly across developmental stages. This spatial structuring of microbial communities within the tick holobiont shapes colonization success, coexistence patterns, persistence, and transmission dynamics.

In ticks, as in other arthropods, the presence of symbionts, commensals, and TBPs is associated with changes in physiology and vector competence, suggesting potential interactions among them [19]. During colonization, pathogens often employ strategies similar to those of symbionts to manipulate the immune response, facilitating their multiplication and transmission [18]. Once established, these pathogens may engage in ecological associations with co-infecting pathogens and the resident microbial community, as inferred from co-detection patterns and community assembly studies [20,21]. These complex interactions occur within a permissive interface, encompassing the host, vector, pathogens, and resident microbial community, and critically determine the success of colonization and subsequent transmission. Structurally and functionally distinct tick tissues generate spatially heterogeneous niches. As a result, the microbial communities residing within the tick holobiont are distributed in a tissue-specific manner, creating a microbial microecosystem in which community assembly and interactions are shaped by local ecological filters (Figure 1).

This means that each tissue (gut, salivary glands, and ovaries) imposes different physiological conditions, immune pressures, and nutrient availability on its resident microorganisms. Consequently, each compartment harbors a unique set of taxa that form local networks of potential metabolic associations, including cross-feeding, competition for resources, and signaling, as suggested by co-localization patterns and in vitro studies [22,23]. This spatial segregation allows the resident gut microbiota to interact directly with pathogens acquired during blood ingestion, whereas the community in the salivary glands becomes more relevant during transmission to a vertebrate host. Furthermore, microorganisms acquired or exchanged at host–vector and environment–vector interfaces can generate local and specific effects within each niche [24].

Within this tissue-structured environment lies the tick microbiome inherent to the tick, comprising bacteria, archaea, fungi, and viruses, both those considered TBPs and those that are not, which must establish itself and exploit the available resources within the vector’s tissues [15]. Their spatial organization and metabolic activities create the ecological context in which the microorganisms, including viruses, must establish themselves and persist. The principles governing these associations extend to the viral components of the holobiont—namely, tick-borne viruses (TBVs), viruses not considered TBPs, and bacteriophages that infect bacterial members of the community—albeit with distinct ecological modalities. TBVs and intracellular bacteria may establish ecological competition within shared cellular niches, determined by the local availability of resources [25]. This competition can manifest at multiple levels: through overlapping tissue tropism, co-localization within the same cells, or metabolic reprogramming of the host cell by one microorganism that alters resource availability for the other [25,26,27].

Many bacteria and fungi within this community can form biofilms, creating structured microenvironments that facilitate resource capture and provide protection against environmental fluctuations and the vector’s immune responses [22]. Bacteria are the most studied component of this resident community [28,29] and are known to form biofilms [30]; they have a complex, structured assembly that may also include fungi [31] and viruses [32] trapped within their matrix. Biofilm-like structures have been observed in tick tissues [22], although direct in vivo evidence of mature biofilms remains limited. The entrapment of viruses within biofilms creates additional opportunities for virus–bacteria interaction [32,33]. Bacteriophages, in particular, are intrinsically linked to the spatial distribution of their bacterial hosts [34,35]. Ecological principles predict that they exert tissue-specific control over bacterial abundance and metabolic activity, although empirical evidence in ticks is still scarce [36]. Thus, the architectural framework of the tick holobiont structures not only the bacterial communities but also the viral populations (both TBVs and phages) that share its cellular environment.

Among this resident community, multiple levels of ecological association are likely to occur among its members, ranging from metabolic cooperation to competition for niches and nutrients. These microorganisms are subject to selective pressures imposed by both the vector’s internal physiology (immune responses, oxidative stress, molting, and blood digestion) and external environmental factors (temperature, humidity, and host factors) [18].

These communities may exhibit functional traits analogous to those observed in other ecosystems, including microbial communication, nutrient cycling, and metabolite exchange. In these established communities, ecological processes such as the loss of taxa and the invasion of new microorganisms can occur, leading to primary or secondary succession events that reconfigure the community’s structure and function over time. Consequently, the complex network of ecological associations within this resident microbial community could largely determine the success of subsequent colonization and transmission of TBPs. Understanding how these factors operate within the tick’s compartmentalized architecture provides the necessary ecological framework to dissect the nature and consequences of microbial interactions, which we will explore in subsequent sections.

## 3. Pathogen–Pathogen Interactions in the Context of Co-Infections

Pathogen–pathogen interactions can be analyzed in terms of the establishment of co-infections. This approach involves analyzing the relationships between pathogens and their implications for virulence and disease dynamics [21]. Co-infections are defined as combinations or assemblies of different pathogens that interact with each other within the host and vector [37]. Microorganisms with structural or functional similarities, which play specific roles within an ecosystem, are grouped into guilds. In the context of pathogen–pathogen interactions, guilds include different species that coexist and may compete for the same resources within hosts or vectors. Pathogens within the same guild can establish synergistic, antagonistic, or neutral interactions that determine virulence, pathogenicity, and colonization success [38,39].

The identification of multiple infections within the vector may suggest an absence of microbial interference between pathogens and the establishment of a possible neutrality or synergistic interaction, facilitating the colonization process [18,40,41,42,43]. However, co-detection alone does not demonstrate direct ecological interaction, as shared occurrence may also reflect common exposure routes, similar ecological requirements, or host-mediated factors.

### 3.1. Insights from Simultaneous Pathogen Detection

In hard ticks (Ixodidae), the coexistence of different *Borrelia* and *Rickettsia* species in co-infection is frequently documented across diverse geographical regions. For example, Banović et al. [44] detected co-infections with *Borrelia lusitaniae* and *Rickettsia helvetica* in *Ixodes ricinus* ticks collected from humans. Similarly, Milhano et al. [45] reported simultaneous infection with *R. helvetica* and *B. lusitaniae* as well as with *R. slovaca* and *B. lusitaniae* in ticks questing *I. ricinus* and *Dermacentor marginatus*. Bertolotti et al. [46] confirmed the predominance of *B. lusitaniae* in the Mediterranean Basin and documented the infection of *I. ricinus* by spotted fever group (SFG) rickettsiae, establishing the ecological context for possible co-infections.

While co-detection alone does not demonstrate ecological interaction, these patterns gain ecological meaning when interpreted within the tick’s compartmentalized architecture. The successful establishment of co-infection between *B. lusitaniae* and *Rickettsia* species in these ticks can be attributed to differences in tropism and the specific pathogenesis mechanisms employed by each bacterium in the tick and vertebrate host. In fact, studies on the localization of pathogens in tick tissues have revealed that these microorganisms can occupy different spatial niches, which could reduce direct competition and facilitate coexistence [47,48]. This specific tissue segregation, a direct consequence of the tick’s structural and functional compartmentalization, allows pathogens to establish themselves in specific compartments. By occupying separate compartments, co-infecting pathogens can avoid interference, and in some cases, local effects in one tissue can generate domino effects that indirectly benefit another pathogen in a distant compartment. This spatial segregation, combined with differential tissue colonization strategies, can create opportunities for simultaneous establishment without interference, supporting the idea of a neutral or even synergistic interaction.

Interestingly, one might expect that similar metabolic needs and ecological niches would lead to competitive exclusion in all cases. However, the coexistence of closely related species within the same bacterial genus that co-infect the same tick may reflect the establishment of synergistic or facilitative interactions. Recent evidence has demonstrated the existence of co-infection within the genus *Rickettsia*, suggesting complex interactions with potentially synergistic outcomes. The traditional view holds that a tick cannot stably maintain two or more different *Rickettsia* species across generations [49]. The primary reason for this may be competitive exclusion or interference mechanisms that prevent a second species from becoming established or being transmitted to the offspring. Despite the robustness of these studies, recent findings based on molecular detection have documented the coexistence of two or more *Rickettsia* species in a single tick. Current evidence indicates that although competitive exclusion is common, particularly under laboratory conditions and with specific species combinations, co-infection may still be possible [49]. This coexistence may be mediated by genetic variability among strains, as well as environmental factors or vector-related traits that modulate the nature and outcome of microbial interactions.

In the genus *Borrelia*, the coexistence of multiple genospecies within the same tick has been widely documented; however, the nature of their interactions remains poorly understood. Moutailler et al. [50] proposed that the presence or combination of some *Borrelia* species may favor the colonization of others within the same genus. For instance, the co-detection of *Borrelia* species in *I. ricinus* ticks [51,52] suggests that the presence of one species does not negatively interfere with the replication of the other, potentially reflecting genotypic heterogeneity, differential tissue tropism or shared amplifying hosts.

Notably, accumulating evidence suggests that the occurrence of synergy, absence of interference, or facilitation among TBPs is primarily driven by the intrinsic properties of the tick vector itself. Studies comparing pathogen prevalence in questing versus feeding ticks have revealed that co-infections occur in both ecological contexts, indicating that the mechanisms enabling coexistence are not merely a consequence of host-derived acquisition but rather reflect the intrinsic properties of the tick holobiont [24]. Vector-related factors, such as physiology, immune tolerance, and microbial community structure, create a permissive environment in which certain combinations of pathogens can coexist in a tick-facilitated environment. The consistent detection of co-infection patterns further reinforces the idea that the vector’s internal ecology plays a fundamental role in shaping these microbial associations.

The interpretational challenges discussed throughout this section are particularly evident when considering virus–bacteria interactions. Experimental studies in tick cell lines have shown that bacterial pathogens can have a positive effect on viral replication under controlled conditions, suggesting facilitative interactions [25]. However, the ecological context in natural conditions is much more complex. In vitro systems lack the ecological complexity of the tick holobiont. The ecological realities could shift the balance from facilitation toward competition, particularly when pathogens (TBVs and tick-borne intracellular bacteria) share the same cellular niches. For example, ultrastructural evidence from naturally infected ticks shows that tick-borne encephalitis virus (TBEV) and *Ehrlichia muris* co-localize in salivary gland cells with cytopathic effects suggestive of resource competition [53]. Therefore, the outcome of virus–bacteria interactions in vivo likely reflects more competitive pressures imposed by the structured environment and resource limitations of the tick holobiont and less direct facilitating mechanisms.

### 3.2. Insights from Pathogen Assembly Patterns Within Guilds

It is important to note that network analysis based on co-occurrence data identifies statistical associations between taxa, not direct ecological interactions. Positive associations may indicate cooperative interactions, shared niche preferences, or similar environmental requirements, whereas negative associations may reflect competition, amensalism, or differential responses to environmental factors. Throughout this section, we use the term ‘association’ to describe these statistical patterns and reserve ‘interaction’ for cases where mechanistic evidence supports a direct ecological relationship.

As exemplified above, the simultaneous detection of pathogens provides valuable information on which microorganisms coexist within ticks. However, defining whether co-infected pathogens engage in synergistic, antagonistic, or neutral relationships requires an analysis of their assembly patterns within ecological guilds. Biological network models provide graphical representations of these pathogen–pathogen association patterns. Recent studies have employed Yule’s Q statistic to construct pairwise pathogen co-occurrence matrices, followed by network analysis, to evaluate the co-occurrence dynamics between TBPs within hosts and ticks and discern patterns of co-occurrence or mutual exclusion [54,55].

A comprehensive investigation of pathogen associations within guilds was conducted by Abdelali et al. [55] in *Hyalomma excavatum* ticks collected from cattle in the Algerian steppe. Using high-throughput microfluidic data from 166 ticks (94 males and 72 females), this study revealed that pathogens are commonly present in guilds and that both abiotic and biotic factors significantly influence their occurrence patterns and associations. Female ticks showed a higher infection rate with common pathogens such as *Rickettsia slovaca*, unclassified Apicomplexa, and *Borrelia afzelii*. Conversely, male ticks showed a lower infection rate, with *Rickettsia* and *R. slovaca* being the most prevalent species. These observations demonstrate the influence of tick sex as a biotic ecological factor on the composition of the pathogen guilds. Notable pathogen–pathogen associations within the guilds were identified through network analysis. Positive associations, such as those observed between *R. slovaca* and *Rickettsia conorii* in males and between *B. afzelii* and *Borrelia spielmanii* in females, are consistent with the previously mentioned evidence of simultaneous detection. These patterns suggest that closely related species within the same genus can coexist under certain ecological conditions, potentially reflecting synergistic or facilitative associations.

**Figure 2:**
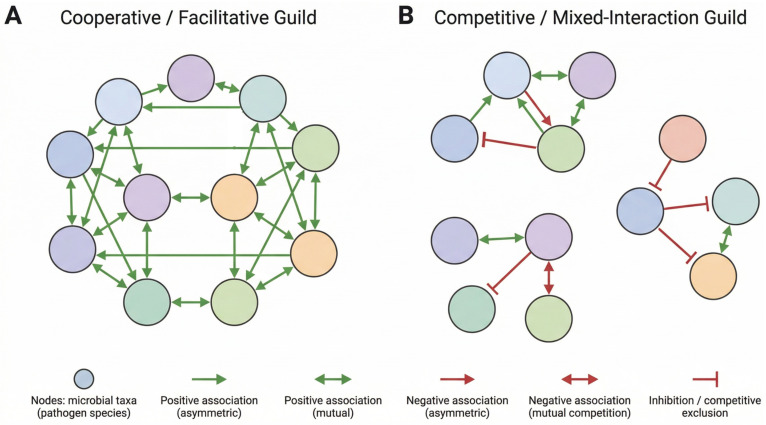
**Guild network dynamics and pathogen–pathogen interaction patterns.** Nodes represent microbial taxa (pathogen species). Edges represent statistically inferred associations derived from co-occurrence network analyses; unidirectional arrows indicate asymmetric interactions, bidirectional arrows indicate symmetric or mutual interactions. (**A**) Cooperative or facilitative guild structure dominated by positive associations (green). Such networks exhibit high cohesion and structural stability, potentially enhancing collective persistence of guild members but limiting colonization by closely related or external taxa (community resistance). (**B**) Competitive or mixed-interaction guild structure characterized by negative associations (red) (e.g., patterns consistent with competitive exclusion) and reduced cohesion. These networks may display lower internal stability but increased permeability to phylogenetically distant invaders. The balance between positive and negative association patterns influences guild stability, susceptibility to invasion, and ultimately pathogen maintenance and transmission within the tick holobiont.

The nature and balance of these associations within a guild have profound implications for its stability and ecological function. A guild dominated by positive associations may reflect cooperative or facilitative relationships that enhance collective persistence within the tick holobiont. This stabilized guild structure could, in turn, positively influence the establishment and subsequent transmission of its constituent pathogens by creating a permissive internal environment that favors their maintenance. However, this stability comes at an ecological cost. A highly cohesive guild with a predominance of positive interactions can become resistant to the establishment and colonization of novel or phylogenetically distinct pathogens. A strong, positively interconnected pathogen guild could function as an exclusive consortium, exhibiting resistance to colonization by non-member pathogens. This phenomenon mirrors the concept of community resistance observed in other microbial ecosystems, in which an established and stable community prevents invasion by external taxa. These contrasting guild configurations and their implications for stability and invasion dynamics are conceptually illustrated in Figure 2.

Interestingly, Abdelali et al. [55] demonstrated that negative associations, such as those observed between *Anaplasma phagocytophilum* and *Francisella tularensis*, were consistent with competitive exclusion. This finding is particularly interesting because both pathogens, despite lacking structural similarities or directly related pathogenesis mechanisms, could compete for overlapping niches within the vector, or their negative association could be mediated by changes in the resident microbiota induced by either pathogen. This observation underscores that antagonism can arise not only from direct confrontation but also from the complex competitive dynamics for resources and space within the tick holobiont. Furthermore, the presence of these negative associations within a guild could generate instability, potentially limiting the coexistence of certain pathogen combinations and shaping the overall structure of the pathogen community in the host. However, a guild destabilized by a higher proportion of negative interactions may become more susceptible to colonization by phylogenetically distant taxa that did not originally belong to the guild. In this alternative configuration, lower internal cohesion may increase community permeability, thereby facilitating colonization by phylogenetically distant pathogens. Therefore, the balance between positive and negative associations within a pathogen guild not only determines its internal stability but also dictates its permeability to external invaders, promoting the exclusion of closely related taxa while potentially opening niches for more distant microorganisms to invade.

## 4. General Biotic and Abiotic Factors Shaping the Tick Microbiome

The tick microbiome is influenced by both biotic and abiotic factors that determine the biodiversity, composition, and relative abundance of its constituent taxa [56,57,58,59]. These parameters are fundamental as they define the ecological space available for interactions: who is present, in what proportions, and with what metabolic or functional potential. Consequently, these three parameters define the configuration of the microbiome as a whole. Therefore, ecological factors can exert two types of influence on microbial community dynamics: an indirect influence by modulating biodiversity, composition, and relative abundance, and a direct influence on the association patterns themselves, independent of the community structure.

### 4.1. Biodiversity, Composition, and Relative Abundance: Community Parameters Shaped by Ecological Factors

Biotic factors intrinsic to the vector play a central role in shaping the microbiome. Comparative studies have demonstrated that tick species, sex, and life stage are important biotic determinants of microbiome dynamics [19,60,61]. Chandra & Šlapeta [62] analyzed the influence of both tick species and life stages by examining the bacterial diversity and composition in *Haemaphysalis bancrofti*, *Ixodes holocyclus*, *Ixodes trichosuri*, and *Ixodes tasmani*, revealing considerable differences between species and life stages. Other studies have demonstrated variations in the microbiome between genera and species within the same genus [63,64]. Sex also contributes to this variation. Van Treuren et al. [64] analyzed the microbiome of adult *Ixodes scapularis* and *Ixodes affinis* ticks collected from 19 locations and found that the microbiomes of females were significantly less diverse than those of males.

Abiotic factors, particularly those related to the external environment, shape tick microbial communities. Geographic location exerts a strong influence; Van Treuren et al. [64] observed that microbiome differences increased with distance between collection sites, with the genera *Rickettsia* and *Borrelia* predominating across all locations. Time is another determining factor. Lejal et al. [58] evaluated temporal dynamics in *I. ricinus* ticks collected over three consecutive years in a peri-urban forest in France, revealing temporal variations among samples collected during different months.

These studies demonstrate that the tick microbiome is a dynamic system that evolves in close relation to the host and environment [3]. Together, these biotic and abiotic factors interact to shape the diversity, composition, and relative abundance of tick-associated microbial communities, establishing the ecological baseline upon which subsequent pathogen-driven selection will act.

The biodiversity, composition, and relative abundance of a tick’s microbial community are not static variables. Communities with high biodiversity, particularly those harboring phylogenetically diverse taxa that employ distinct resource exploitation strategies, could theoretically exhibit greater stability [65,66,67,68]. Metabolic differences in resource exploitation reduce competition. In this sense, cooperative interactions, such as cross-feeding, can be facilitated, where the metabolic by products of one taxon become substrates for another, potentially promoting the growth of some community members at the expense of others [69,70]. However, phylogenetic and metabolic diversity do not always lead to peaceful coexistence. Differences in resource exploitation pathways can result in interference mechanisms that actively inhibit the growth of competing taxa, including the production of antimicrobial secondary metabolites (e.g., bacteriocins) and extracellular lytic enzymes [71]. This form of interference competition can increase ecological tension within a community, even in the absence of direct resource overlap. Therefore, the same parameters that define community structure also condition the balance between cooperative and competitive forces.

### 4.2. Community Assembly: The Ecological Processes Underlying Community Structure

The biodiversity, composition, and relative abundance modulated by these factors are not static endpoints. These are observable outcomes of the underlying community assembly processes. In community ecology, assembly refers to the processes that govern the establishment, persistence, and interaction of microorganisms over time [2,72]. Extrapolating to the tick-borne transmission environment, these processes could include dispersal, conceptualized as the arrival of microorganisms from external sources (environment and hosts) or internal reservoirs (vertical transmission). Environmental selection acts as a filter imposed by the tick’s physiology, its immune response, and specific tissue conditions. Ecological drift encompasses random changes in microbial populations, a process particularly relevant during the extended off-host periods that characterize tick life cycles, when prolonged starvation may amplify stochastic fluctuations in community composition. Finally, diversification involves evolutionary changes within the community over time.

Biotic and abiotic factors modulate assembly by affecting microbial growth, potential interaction patterns, and the balance between cooperative and competitive associations [3]. Taxa within the community form cooperative or competitive associations that can facilitate or hinder subsequent colonization by both pathogenic and non-pathogenic microorganisms. In this regard, co-occurrence network approaches have been used to analyze community assembly and predict, in silico, the response to external perturbations [73]. Previous comparisons of bacterial assembly in hard ticks have revealed a predominance of positive associations among taxa [73]. This pattern, which may reflect cooperative relationships, could be explained by the abundance of taxa with diverse metabolic requirements, which reduces competition for nutrient availability. In other microbial systems, cooperation among microorganisms depends on the diversity of metabolic requirements, genotypic and phenotypic differences between species, and the carrying capacity of the environment [7,74,75,76]. Whether similar mechanisms operate within the tick holobiont remains to be tested experimentally.

Microbial communities may exhibit resistance (the ability to withstand disturbance) or resilience (the capacity to recover following perturbation) in response to external perturbations [77]. Tick microbial communities likely display similar behaviors. The tick’s life stage can influence this response. Recent in silico manipulation studies of *Rhipicephalus microplus* have revealed differences in network robustness between nymphal and adult stages, suggesting that resilience mechanisms are stage-specific [78].

At the network level, robustness reflects the preservation of structural and functional integrity despite the loss or addition of taxa. In silico approaches, such as node removal or addition, allow for the assessment of network robustness. These analyses provide insights into how ecological processes, such as displacement or microbial invasion, can affect community stability.

This dual potential reinforces the previously presented idea that ecological factors exert two distinct types of influence on the microbial community structure and dynamics within ticks. First, an indirect influence operates by modulating biodiversity, composition, and relative abundance, thereby determining the set of potential interactors. Second, a direct influence acts on the association patterns themselves. Understanding how these two levels of influence operate in the tick holobiont is essential for predicting how the resident community will respond to the arrival of TBPs.

The community assembly processes (dispersal, selection, drift, and diversification) offer a useful perspective for formulating questions about the effects of pathogens. When a tick-borne pathogen becomes established within the holobiont, it can not only add a new taxon to an existing assembly but also alter the relative contribution of each assembly process to community dynamics. For example, pathogens could modify environmental selection by manipulating immune responses, affect dispersal by competing with incoming microorganisms, or influence ecological drift by altering the population size of resident taxa. Distinguishing these possibilities experimentally remains a challenge, but formulating them as hypotheses is a necessary first step in this process. This perspective provides a conceptual basis for the dual selective pressure framework that will be developed in the next section, while also highlighting where mechanistic evidence is still needed.

## 5. Pathogen–Non-Pathogenic Microbial Community Interactions

The microbial community associated with ticks, like any other ecosystem, coexists in a dynamic environment subject to selective pressures that determine the balance between cooperative and competitive associations among its members [3,12,75]. A diverse and stable microbial community can exhibit resistance to colonization and expansion by potential pathogens. However, external perturbations can alter this balance, reduce resistance to colonization, and create opportunities for pathogen establishment [79].

To understand the following section, the term “absence of pathogen-induced selection” requires clarification. Similar to other ecosystems, the tick microbial community may include members that act as vertebrate pathogens or opportunistic taxa under certain conditions, but this does not equate to the selective pressure exerted by TBPs within the tick. The latter involves active replication, resource consumption, and immune modulation, all of which facilitate subsequent transmission.

Throughout this section, we will refer to the “non-pathogenic microbial community” as an operational term encompassing all microorganisms that are not considered TBPs; that is, those that are not part of the group of pathogens that complete their transmission cycle via ticks. This definition deliberately includes symbionts, commensals, environmentally acquired microbes, and even members that can be vertebrate pathogens or opportunistic under specific circumstances. The reason is that their potential to cause disease outside the tick does not translate into the intravectorial selective pressure exerted by actively replicating TBPs. Therefore, the non-pathogenic community, as defined here, represents the entire ecological context in which TBPs establish and interact.

The following sections first distinguish which factors operate independently of pathogen-driven selection and then examine how the dual selective pressure imposed by basal factors and replicating pathogens jointly shapes microbial communities and their interactions.

### 5.1. Factors That Operate in the Absence of Pathogen-Driven Selection

In the previous section, the general biotic and abiotic factors that shape the tick microbiome and, consequently, its microbial interactions were examined. However, these factors are usually studied in ticks collected from the field, where exposure to pathogens cannot be ruled out. Next, we will address which of these factors operate independently of tick-borne pathogen-induced selection. The tick microbial community is active in its basal state, that is, in the absence of selective pressure imposed by replicating TBPs. Understanding this basal behavior will allow us to distinguish between the intrinsic dynamics of the community and the disturbances introduced by pathogens once they become established on the vector.

Studies on the soft ticks *Ornithodoros erraticus* and *Ornithodoros moubata* under pathogen-free conditions provide direct evidence of how the tick microbiome functions in the absence of pathogen-induced selection [80]. By minimizing covariates such as stage, engorgement, and host, these studies demonstrated two fundamental characteristics of the tick holobiont. First, they confirmed compartmentalization: microbial communities are tissue-specific, with differential assembly in the midgut and salivary glands. This tissue specificity persists without pathogen pressure, indicating that each compartment imposes its own ecological filters. Second, they showed that tick physiology shapes microbial diversity and community structure. Differences in community assembly among species were related to variations in the transcriptome, anatomy, and midgut physiology, highlighting that intrinsic vector traits act as selective forces independent of pathogens [81,82]. Notably, these findings reveal a relationship contrary to the usual one between biodiversity and competition. In *O. erraticus*, lower midgut diversity was associated with greater microbial competition, which could favor the survival of better-adapted taxa and closer associations among the remaining community members. This competitive restructuring could generate a community that is hypothetically more resistant to pathogen colonization. In contrast, *O. moubata* harbored a less competitive and potentially more permissive microbiota. While experimental studies are needed, these patterns could explain why *O. moubata* is more competent than *O. erraticus* at transmitting African swine fever virus and tick-borne relapsing fever spirochetes.

Studies of laboratory-reared hard tick colonies offer further evidence of factors that operate independently of pathogen-induced pressure. Kamani et al. [83] compared adult females and their laboratory-reared larval offspring in two tick species: *Rhipicephalus linnaei* and *Haemaphysalis leachi*. Although adult ticks were collected from dogs in Nigeria and could have been exposed to pathogens, their unfed larvae (reared in the laboratory without blood feeding) offer a window into the vertical transmission component of the microbiome. The presence of *Arsenophonus* and *Coxiella* in both adults and larvae confirms that these symbionts are maintained across generations through vertical transmission, a process that operates independently of pathogen pressures. Furthermore, significant differences in alpha and beta diversity between species and life stages demonstrate that tick species identity and developmental stage are intrinsic determinants of microbial community structure, even when environmental acquisition is limited.

Taken together, these examples illustrate a broader principle: in the absence of pathogens, the tick microbiome is structured by species, tissue, and physiologically determined factors, as well as continuously operating vertical transmission processes. They also demonstrated that reduced biodiversity can, under certain conditions, enhance community cohesion and potentially influence resistance to invasion, a hypothesis that warrants further experimental testing.

### 5.2. Dual Selective Pressure: How Pathogens and Basal Factors Jointly Shape Microbial Communities and Their Interactions

Basal factors function as ecological filters that determine which taxa can establish themselves and persist in each niche, as well as the assembly of the community itself. In contrast, pathogens are active modifiers of the filters themselves; thus, pathogen-driven selection constitutes a qualitatively distinct force. Through immune modulation, nutrient depletion, biofilm disruption, and other mechanisms, replicating pathogens can alter the tick’s physiological environment, thereby modifying the selective pressures acting on all members of the community.

The ecological impact of *Anaplasma marginale* on the *R. microplus* microbiome provides an example of how pathogens exert selective pressure that, superimposed on the baseline temporal dynamics, reconfigures microbial communities [84]. Analysis of infected ticks at three time points, while minimizing covariates such as sex, life stage, and host, revealed that microbial diversity decreased as the relative abundance of *A. marginale* increased. Significant changes in diversity, composition, and abundance were observed over the years, indicating that the *R. microplus* microbiome is transient and sensitive to pathogen pressure. From an ecological perspective, this relationship is bidirectional; low biodiversity could facilitate the establishment of pathogens by reducing community resistance [85,86]. However, in this case, the pathogen itself could drive the loss of diversity, creating a feedback loop that favors its own persistence.

Co-occurrence network analysis has shown that colonization by *A. marginale* reconfigures community assembly [84]. Over time, the number of microbial correlations decreased, as did modularity, indicating fragmentation of the community into less interconnected modules. Most of the remaining associations were positive, which may reflect a shift toward cooperative relationships among surviving taxa. Notably, *A. marginale* transitioned from a peripheral participant to a keystone taxon by the end of the timeline, meeting established criteria [87,88]: it was ubiquitously present across samples, exhibited high eigenvector centrality (>0.75), and showed above-average relative abundance [84]. This shift was accompanied by the formation of an independent module with positive correlations, suggesting progressive consolidation of the pathogen within the microbial network. Simultaneously, *Rickettsiella*, which exhibited co-exclusion with *Anaplasma*, disappeared over time, a pattern suggestive of competitive displacement as an ecological process favoring pathogen colonization.

This pattern of pathogen-driven fragmentation is not unique to *A. marginale*. A parallel example is found in the interaction between *A. phagocytophilum* and the *I. scapularis* microbiome, where biofilm disruption serves as the mechanistic basis for community reconfiguration [22,89].

Biofilm formation illustrates the interplay between basal factors and pathogen pressure. In the absence of pathogens, bacterial biofilms represent a key cooperative strategy within the tick microbiome. These structured microenvironments facilitate resource sharing, such as cross-feeding and syntrophy, while also providing stability and protection against external perturbations [90,91]. Biofilm-associated interactions enhance community cohesion and resilience, contributing to the basal stability determined by tick physiology and tissue-specific conditions.

However, TBPs have developed mechanisms to disrupt these cooperative networks, adding a layer of pathogen-driven selection. Abraham et al. [22] demonstrated that *A. phagocytophilum* colonization of *I. scapularis* induces the expression of a tick antifreeze glycoprotein, which inhibits biofilm formation and alters the microbiota to favor pathogen establishment. This disruption extends beyond structural changes to affect functional relationships within the community. Functional analyses using PICRUSt predictions revealed that *A. phagocytophilum* infection does not alter the diversity of metabolic pathways but induces changes in their composition, with unique pathways appearing in infected ticks [89]. These changes may reflect a pathogen-driven cascade: *A. phagocytophilum* alters tick physiology, disrupting biofilm structure and triggering a functional reconfiguration of the resident microbial community that ultimately favors its own colonization. However, this linear cascade may not capture the complexity of simultaneous multiple interactions, cross-regulatory effects, and compensatory responses that real biological systems exhibit.

Notably, infections cause fragmentation of metabolic networks. Topological properties, such as modularity, indicate a disruption of metabolic pathways in the infected group, resulting in a more compact but functionally specialized network. This fragmentation is consistent with the inhibition of biofilm formation; as cooperative structures are disrupted by pathogens, metabolic interdependencies, including cross-feeding and syntrophic relationships that sustain the community, are lost. The resulting reconfiguration reduces network resilience, creating a more permissive environment for pathogen colonization.

Examples of *A. marginale* and *A. phagocytophilum* illustrate how pathogen-driven selective pressure operates in ticks. Unlike basal factors, which function as relatively stable ecological filters, pathogens can actively modify these filters through mechanisms such as immune modulation, nutrient competition and biofilm disruption. Superimposed on basal factors, such as temporal dynamics and biofilm-mediated cooperation, this pressure can lead to the fragmentation of microbial networks, loss of diversity, and displacement of competing taxa.

The response of tick microbial communities to dual selective pressures is not uniform. A study on *H. excavatum* ticks from Algeria revealed an alternative outcome: the emergence of keystone taxa that stabilize community structures under changing conditions [92]. Analysis of the microbiome of female ticks collected during three seasons (spring, summer, and autumn) revealed significant seasonal changes in beta diversity, whereas alpha diversity remained unchanged [92]. This indicates that temporal variation, a baseline factor, acts as a selective force that reconfigures microbial assembly without necessarily altering the overall richness and evenness. Superimposed on this temporal baseline, the presence of pathogens such as *Rickettsia* (whose prevalence varied seasonally) further modulated the microbial interactions. Co-occurrence network analysis showed that microbial association patterns changed across seasons, particularly between symbionts and pathogens.

Notably, *Francisella* emerged as a keystone taxon in the network, playing a central role in maintaining community cohesion and buffering the system against pathogen-induced filter modification. Its status as a keystone taxon became evident under specific seasonal conditions, suggesting that dual selective pressure (seasonal variation + presence of pathogens) can cause certain community members to assume a disproportionate functional importance within the network. This finding adds an important dimension to the dual pressure framework: pathogens and basal factors do not always drive communities toward fragmentation and instability. In some contexts, they can promote the emergence of stabilizing elements, such as keystone taxa, that enhance network robustness and preserve community integrity despite the presence of pathogens.

The examples above illustrate how bacterial pathogens and symbionts can either destabilize or stabilize tick microbial communities depending on the ecological context. However, viruses introduce an additional layer of complexity. A recent experimental study on *I. ricinus* nymphs reveals how viruses can produce stabilizing effects under controlled conditions [93]. In this system, larvae fed on mice infected with TBEV, *B. afzelii*, or both, and were allowed to molt into nymphs under pathogen-free conditions without environmental microbial acquisition. Thus, the only selective forces operating during molting were: (i) the basal physiological filter of metamorphosis, which re-establishes the microbial community, and (ii) the replicative pressure of both pathogens acquired during feeding. Under these closed conditions, with dispersal (environmental acquisition) effectively blocked, TBEV infection increased microbiome diversity and improved network robustness compared to uninfected or *Borrelia*-infected nymphs. This stabilizing effect contrasts with the fragmentation observed with *A. marginale* [84] and *A. phagocytophilum* [89] in open systems. The difference may be due to the fact that in a closed system, viral replication can stabilize resident communities by modulating tick immunity or by occupying cellular niches without directly competing with bacteria.

These findings reinforce the dual selective pressure framework by demonstrating that the outcome of pathogen pressure depends critically on the baseline context. In closed systems where dispersal is minimal, viruses can enhance community stability, whereas in open systems with continuous microbial flow, pathogens can contribute to network fragmentation. Therefore, the framework considers both stabilizing and destabilizing outcomes by emphasizing that the effects of pathogens are modulated by the strength and nature of baseline filters.

## 6. Conclusions and Future Perspectives

Ticks should be viewed not as passive carriers of pathogens but as compartmentalized holobiont systems in which microbial communities assemble and interact under defined ecological constraints. By integrating ecological theory with vector biology, this review highlights how pathogen–pathogen and pathogen–microbiome interactions emerge within the spatially heterogeneous architecture of ticks and ultimately shape vector competence.

Three main conclusions arise. First, tissue compartmentalization generates discrete ecological niches that structure microbial communities and influence their coexistence, potential competitive dynamics, and transmission dynamics. Spatial segregation can reduce direct interference, facilitating certain pathogen combinations while limiting others. Second, the pathogen guild structure reflects a balance between positive and negative associations that determine community cohesion, stability, and permeability to invasion. Configurations dominated by positive associations may enhance collective persistence but resist colonization by additional taxa, whereas those with prevalent negative associations may destabilize networks and alter invasion dynamics. Third, microbial assembly within ticks is governed by dual selective pressure: basal ecological factors establish the baseline microbiome, whereas pathogen-driven processes reconfigure community networks through replication, immune modulation, biofilm disruption, and patterns suggestive of competitive displacement.

These interacting forces can lead to alternative outcomes. Biofilm disruption by pathogens can trigger network fragmentation and diversity loss. In contrast, keystone symbionts may buffer the system, promoting greater stability. Alternatively, pathogens that achieve high prevalence without destabilizing the network can themselves become keystone taxa. Identifying the conditions that favor each outcome is a key priority for future research.

Although co-occurrence and network analyses provide valuable insights, they do not alone demonstrate mechanistic interactions. Integrative approaches to studying microbial interactions in ticks (such as simultaneous pathogen detection, microbiome analysis, and predictive network models) now require further reinforcement through complementary strategies.

To move beyond descriptive co-occurrence patterns toward a mechanistic understanding of microbial community dynamics within the tick holobiont, an integrative methodological framework is required. Evolutionary genomics provides a powerful foundation for identifying the genetic determinants associated with adaptation, metabolic specialization, and resistance to host-mediated selective pressures. High-throughput sequencing platforms (e.g., Illumina, PacBio, and Nanopore) enable the detection of genomic variations, whereas phylogenetic and comparative approaches allow the contextualization of these variants within broader evolutionary lineages. Computational evolutionary models can further predict adaptive trajectories under defined ecological conditions.

However, genomic inference alone is insufficient to establish a causative relationship. Experimental validation is essential to disentangle direct microbial interactions and host-mediated effects. Controlled co-infection assays, in vitro biofilm models, and functional approaches such as metabolomics and transcriptomics can reveal mechanistic links between genetic potential and ecological outcome. Time- and tissue-specific sampling designs are particularly important for capturing the spatial and developmental heterogeneity that characterizes microbial communities within ticks. The absence of detailed temporal data in case examples limits understanding of whether observed patterns represent stable states, transient dynamics, or cyclical processes.

In addition, future research should also address which community assembly processes (dispersal, environmental selection, biotic interactions, or ecological drift) are primarily affected by pathogen colonization. The dual selective pressure framework proposed here generates testable hypotheses: for example, do pathogens alter environmental selection by modulating tick immunity, or do they tip the equilibrium toward ecological drift by reducing the populations of resident taxa? Do keystone taxa stabilize communities by reinforcing environmental selection against invaders? Distinguishing these possibilities experimentally will require controlled infections along with time-series sampling, manipulative experiments that disrupt specific assembly processes, and the development of theoretical models that can predict outcomes under different selective regimes. Articulating these questions is a necessary first step in moving from patterns to processes.

Several critical knowledge gaps require special attention. Future research should prioritize studying, in open systems where microbial dispersal operates, (i) the role of bacteriophages in shaping tick-associated bacterial communities, (ii) the impact of viral replication on interactions between TBVs and intracellular bacteria (both pathogens and symbionts), and (iii) the interactions between viruses and the non-pathogenic microbial community (including bacteria, fungi, and archaea).

By integrating evolutionary genomics, predictive modeling, and experimental validation, future research can bridge the gap between ecological theory and molecular mechanisms. Such integration will be critical for understanding how selective pressures shape microbial networks and, ultimately, for designing microbiome-based strategies to reduce vector competence and mitigate tick-borne disease transmission.

## Data Availability

No new data were created or analyzed in this study.
